# Mandibular extension as a trigger of the proprioceptive trigeminocardiac reflex

**DOI:** 10.55730/1300-0144.6131

**Published:** 2025-10-03

**Authors:** Massimo CONTI, Giovanna TRAINA, Daniele TONLORENZI

**Affiliations:** 1 Independent researcher, no affiliation, Carrara, Italy; 2 Division of Food, Biochemical, Physiological and Nutritional Sciences, Department of Pharmaceutical Sciences, Perugia University, Perugia, Italy

**Keywords:** Trigeminocardiac reflex, cerebral vasodilation, mandibular extension, proprioception, autoregulation

## Abstract

The trigeminocardiac reflex (TCR) is a physiological response characterized by a reduction in heart rate and arterial blood pressure following stimulation of any branch of the fifth cranial nerve. This review presents a novel mechanism of TCR induction through proprioceptive activation of the trigeminal nerve via mandibular extension (ME). Accordingly, it is referred to as the “proprioceptive trigeminocardiac reflex” (pTCR). An overview of the existing literature on ME-evoked TCR in humans and animals is presented, with particular emphasis on evidence of cerebral microcirculatory alterations. The magnitude of cardiovascular responses appears to depend on the duration of ME, with repeated applications producing more pronounced and prolonged effects, particularly in hypertensive subjects. In rats, pTCR induction is accompanied by a marked increase in pial arteriolar diameter within the frontal and parietal cortices, apparently independent of neurovascular coupling. Anatomical studies propose a putative neural pathway underlying the pTCR, originating in the mesencephalic trigeminal nucleus and projecting to cardiovascular control centers within the brainstem. Experimental evidence indicates that the TCR may represent a centrally mediated neurogenic reflex resulting in decreased arterial pressure and cerebrovascular vasodilation. A deeper understanding of this reflex may pave the way for developing noninvasive interventions aimed at preventing or delaying the onset of hypertension and stroke, both of which are increasingly prevalent in Western populations.

## 1. Introduction

Painful stimulation of the head, which is innervated by the trigeminal nerve (cranial nerve V), has long been recognized to consistently elicit reductions in blood pressure (BP) and heart rate (HR) [1]. This phenomenon, described in the literature as the trigeminocardiac reflex (TCR), is typically induced by mechanical or chemical stimulation of any branch of the trigeminal nerve [2]. The incidence of this response during ocular and maxillofacial surgery is relatively high and, if not promptly managed, may lead to serious complications such as asystole or even death [3]. In recent years, extension of the mandibular muscles has been shown to induce cardiovascular responses closely resembling those observed in the TCR, in both humans [4] and laboratory rats [5]. Because this procedure activates trigeminal proprioception—specifically through the muscle receptors of the masseter and temporalis—the resulting cardiovascular depression has been termed the “proprioceptive trigeminocardiac reflex” (pTCR). This review provides a comprehensive overview of the existing literature and offers theoretical insights into the physiological significance of the reflex. It also aims to explore potential therapeutic applications and identify key directions for future research. Due to the limited number of available studies, no formal inclusion or exclusion criteria were applied.

## 2. The trigeminocardiac reflex family

The TCR is part of a broader family of reflex reactions that includes the oculocardiac reflex and the diving reflex, often grouped under the term trigeminovagal reflex due to their shared pathway and resulting effects [1]. The oculocardiac reflex has long been observed during eye surgery, consisting of bradycardia and arterial hypotension associated with traction applied to the extraocular muscles and manipulation of or around the eyeball [6]. The diving reflex occurs when the face is immersed in cold water, causing bradycardia and, unlike the TCR, excitation of the vasomotor center followed by an increase in blood pressure [7]. While the TCR and oculocardiac reflex are less clearly defined in their physiological roles, the diving reflex has a well-established function in diving species, namely reducing overall oxygen consumption during submersion and diverting blood to the heart and brain [8]. The pTCR adds a proprioceptive mechanism to this family of reflex cardiovascular responses, which will be discussed in the following section.

### 2.1. The proprioceptive trigeminocardiac reflex (pTCR)

The first description of TCR induced by mandibular extension (ME) was provided by Brunelli et al. [4]. In their study, 18 normotensive university students underwent 10 min of submaximal ME using an oral device consisting of an elastic, U-shaped surgical steel plate. The authors observed significant reductions in systolic and diastolic BP and HR that persisted for 80 min after the end of stretching. The reductions were approximately 10% for BP and 15% for HR. Subsequent studies in humans and laboratory rats have built upon these preliminary findings. Del Seppia et al. [9] replicated the previous findings in healthy volunteers, documenting a 5-mmHg reduction in systolic and diastolic BP that persisted throughout the 120 min observation period. No changes in HR were observed. In a subsequent study by Del Seppia et al. [10], a mean BP reduction of 2.4 mmHg was observed, accompanied by a small but significant decrease in HR. It is important to note that in this experiment, the dilator held each subject’s mouth open at a constant 60% of their maximum opening. In contrast, participants in previous studies were instructed to close their mouths periodically on the device, simulating assisted masticatory movements.

In the animal model, the pTCR was elicited by placing a dilator between the animal’s upper and lower dental arches to achieve maximal mouth opening [5]. Experiments were conducted in male rats (n = 15), whose mean arterial BP and HR were continuously monitored for 80 min after a 10 or 15 min ME. Mean BP and HR decreased by approximately 20% from baseline during ME and remained consistently reduced throughout the entire observation period. These effects were less pronounced when the duration of stretching was limited to 5 min. The response was abolished by cutting the trigeminal nerve. Subsequently, Lapi et al. [11] extended the observation period in 10 rats and found that the reductions in BP and HR persisted for 160 min after the end of ME.

Repeated ME resulted in a more pronounced and prolonged effect in rats, particularly in those with hypertension. Following a single early repetition of mandibular extension (ME), seven normotensive rats showed reductions in BP and HR lasting up to 240 min [12]. In the study by Del Seppia et al. [13], a second ME was administered to five experimentally hypertensive male rats 2.5 h after the first, once their BP had returned to baseline. The resulting reduction in BP was greater than in the first trial, dropping to 25% below baseline. HR also showed a significant decrease of approximately 20%. In the study by Sabatino et al. [14], three spontaneously hypertensive rats showed a 15% decrease in BP—but not in HR—after the second ME, which lasted for the subsequent 270 min observation period. These findings have important implications regarding the modulation of gene expression and protein levels of the renin–angiotensin system and thyroid hormones observed in different regions of the cerebral cortex in relation to ME [15]. These hormones are important regulators of cerebral vasculature and are known to play a role in the pathophysiology of hypertension. A preliminary study by Del Seppia et al. [16] also documented a 10 mmHg decrease in mean BP in a small cohort of seven hypertensive adults subjected to a 10 min ME.

### 2.2. Differences between TCR and pTCR

TCR is usually defined as a heart rate below 60 bpm and a mean arterial blood pressure that is at least 20% lower than baseline. More recently, Meuwly et al. classified TCR into peripheral and central subtypes, depending on whether the stimulus acts on the afferent endings (peripheral) or on the trigeminal ganglion and nuclei (central) [2]. While bradycardia is common to both TCR variants, peripheral TCR does not necessarily involve a decrease in arterial pressure. According to this classification, pTCR belongs to the peripheral subtype, although its cardiovascular effects are generally less pronounced, particularly in humans. Gastric hypermotility is another typical reaction elicited by TCR that is not observed in pTCR. Cardiovascular changes are typically reversible after removal of the stimulus that evoked the TCR. Furthermore, as noted by Schaller et al. [6], the TCR weakens with repeated stimulation, whereas its proprioceptive variant appears to strengthen after repeated ME, reaching its maximum effect several 10 min after the stimulus has ceased [12–15].

TCR and related reflexes pose a serious threat to patients undergoing medical procedures, potentially leading to severe arrhythmias, asystole, or even death [17]. Preventive and management strategies are thus of crucial importance, especially for patients with cardiovascular disease. In contrast, the slow onset of pTCR, combined with its milder effects, renders it an almost entirely benign condition. From a clinical perspective, the decrease in heart rate and arterial pressure that follows pTCR induction suggests its potential as a therapeutic approach for managing hypertension and anxiety-related cardiovascular symptoms.

### 2.3. Neuroanatomical aspects

The neuroanatomical basis of the trigeminocardiac reflex has been determined through animal research. Trigeminal sensory afferents reaching the Gasserian ganglion project to the brainstem. The spinal nucleus of the trigeminal nerve, located in the medulla, represents the first relay station in the afferent arc of the reflex [1]. Neurophysiological evidence indicates that fibers from the trigeminal nuclei indirectly reach the nucleus ambiguus in the medullary reticular formation, which, together with the dorsal motor nucleus of the vagus, sends inhibitory output to the heart [18]. Gorini and colleagues demonstrated in rats that this pathway is differentially modulated by serotonin receptors and inhibited by the action of a muscarinic agonist [19,20].

The pTCR likely relies on the same central pathways as the exteroceptive TCR, as illustrated in the Figure; however, direct physiological evidence remains lacking. The afferent input for the pTCR is presumed to originate from proprioceptive fibers innervating the trigeminal mesencephalic nucleus (MeV) located in the pons. MeV neurons are first-order cells that convey proprioceptive signals from the jaw muscles and the periodontium [21]. In animals, these neurons exhibit extensive projections to numerous brainstem nuclei, including the dorsolateral reticular formation, and also innervate the cerebellum and spinal cord [22,23]. Using horseradish peroxidase (HRP) labeling, Kubota et al. [24] also demonstrated a direct MeV projection to the dorsal motor nucleus of the vagus in mice.

## 3. Modulation of cerebral blood flow

In their study, Lapi et al. [5] also found that mandibular extension increases cerebral perfusion in rats. Application of the mouth-opening device for 10 min resulted in sustained dilation of pial arterioles in the left parietal cortex, lasting for approximately 80 min. This effect was more pronounced in small vessels. However, no changes were observed in rats subjected to bilateral trigeminal nerve transection.

These findings were later replicated in subsequent studies. Lapi et al. [11] reported that the vasodilatory effect of ME persisted for up to 3 h following a 10 min application. A shorter duration (5 min) resulted in a more transient effect, with the response subsiding after only 40 min. Evidence suggests that the vasodilation was mediated by nitric oxide (NO), given that a nitric oxide synthase (NOS) inhibitor abolished the effect and that neuronal and epithelial NOS expression were upregulated during and after ME. Early repetition of ME significantly prolonged the vasodilatory effect, which lasted for more than 240 min after the initial mandibular extension. Moreover, spectral analysis showed that ME modulated the intrinsic rhythmic vasomotion pattern of pial arterioles by increasing the amplitude of low-frequency oscillations (related to endothelial activity) and reducing the highest-frequency components. Lapi et al. [25] expanded these observations to artificially induced hypertensive rats. Another key finding was the observation of ME-induced vasodilation within the left frontal cortex. This result has significant implications, as it demonstrates that the vasodilatory effects of ME are not confined to regions directly innervated by trigeminal afferents. Similarly, Goadsby and Duckworth [26] previously observed an increase in cerebral blood flow (CBF) within the parietal and frontal regions of the brain following stimulation of the trigeminal ganglion. This effect was found to be blocked by bilateral facial nerve section.

### 3.1. Mechanisms underlying changes in cerebral blood flow (CBF)

Changes in pial arteriolar diameter are normally linked to neurovascular coupling, i.e. an increase in localized CBF following neural activation [27]. For example, dilation has been reported in upstream pial arterioles of the rat brain after neuronal activation of the somatosensory cortex by whisker stimulation [28]. Nonetheless, neurovascular coupling appears insufficient to explain the prolonged vasodilatory effect of mandibular extension. In fact, experiments described above consistently revealed a brief, unexpected constriction of cerebral arterioles during ME itself. As demonstrated by Lapi et al. [11], this effect is mediated by opioid receptors, as it is completely blocked by the opioid receptor antagonist naloxone. This evidence suggests that trigeminal stimulation directly influences vasoconstriction. Reflex autoregulatory mechanisms, designed to maintain a constant CBF despite blood pressure fluctuations, are also unlikely to play a significant role. This is because the observed vasodilation appeared to be, at least partially, independent of BP reduction, persisting for more than 20 min after BP returned to baseline levels.

To account for the observed changes—initial vasoconstriction during ME followed by persistent vasodilation independent of BP changes—multiple mechanisms are likely to interact. In the past, several studies have reported changes in cerebral perfusion pressure and/or vascular resistance following nonproprioceptive trigeminal stimulation. Whether trigeminal nerve stimulation leads to an increase [29] or a decrease [30] in CBF appears to depend on the nature and characteristics of the stimulation and the particular vascular bed being considered. Proposed mechanisms include cross-stimulation between trigeminal and parasympathetic fibers running through the seventh cranial nerve and the antidromic release of vasodilatory neuropeptides from trigeminal perivascular endings [31]. Some researchers have suggested the involvement of the rostral ventrolateral medulla (RVLM), a vasomotor center containing oxygen-sensitive neurons. The trigeminal ganglion sends direct projections to the RVLM, which is presumably activated by stimulation of the exteroceptive TCR [32]. Through a route that is not well characterized, the RVLM is thought to project to intracortical vessels [33,34].

The TCR highlights a close and not yet fully understood connection between the trigeminal system and the intricate regulation of cerebral blood flow, one that extends beyond the well-known mechanisms of neurovascular coupling and autoregulation.

### 3.2. The “brain emergency system”

Taken together, TCR-induced changes may represent a mechanism that regulates cerebral blood flow in response to emergency situations. This aligns with Schaller’s [1] suggestion that the TCR may represent a physiological entity serving an oxygen-conservation function. It is possible that trigeminal-mediated vasodilation plays a compensatory role when autoregulation fails. For example, Moskowitz et al. [35] found that the trigeminal nerve promoted pial vasodilation during acute hypertension and pharmacologically induced vasoconstriction. Moreover, electrical stimulation of the trigeminal nerve has been shown to reduce lesion volume following traumatic brain injury [36]. In rats subjected to hemorrhagic shock, trigeminal stimulation induced a series of changes, including an increase in CBF, which prolonged survival in the short term [37]. In many forms of senile dementia, reduced vascular perfusion deprives specific brain regions of oxygen and nutrients, leading to progressive and irreversible neuronal loss. This, in turn, contributes to cognitive decline and a general deterioration in quality of life [38]. These observations highlight the therapeutic potential of targeting trigeminal pathways to improve cerebral blood flow and preserve neurological function.

## Conclusions

Experimental evidence suggests that the TCR may represent a centrally mediated neurogenic reflex, resulting in decreased arterial pressure and cerebrovascular vasodilation. Based on these findings, it is plausible to consider proprioceptive stimulation of the trigeminal nerve (pTCR) as a target for developing noninvasive procedures aimed at preventing or delaying the onset of medical conditions such as hypertension, dementia, and cerebral stroke, which are prevalent in Western populations.

However, several practical challenges and limitations must be addressed. Current research is largely based on studies with small sample sizes, making it difficult to draw definitive clinical conclusions. Moreover, the lack of standardized protocols for stimulus application and duration poses a challenge for reproducibility and clinical implementation. Future research should focus on validating the observed effects in larger populations and on developing a reliable and reproducible therapeutic protocol. Furthermore, additional studies are needed to better understand the long-term effects and safety of repeated pTCR, and to fully elucidate its neuroanatomical pathways in both animals and humans.

## Figures and Tables

**Figure:**
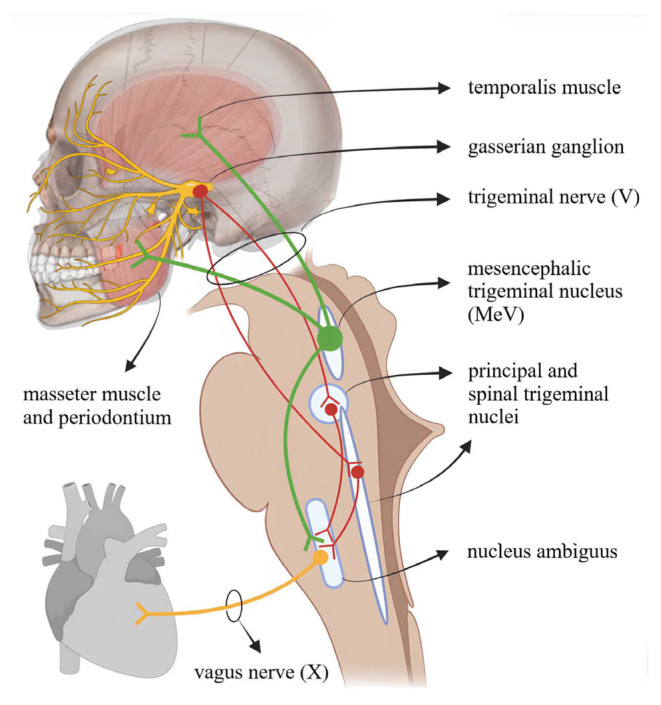
Anatomical pathways of the TCR (red) and its pTCR variant (green) that converge on the nucleus ambiguus. Intermediate neural relays are omitted for clarity. (Figure created with https://BioRender.com ).

## References

[1] Schaller B Trigeminocardiac reflex. A clinical phenomenon or a new physiological entity? Journal of Neurology 2004 251 6 658 665 10.1007/s00415-004-0458-4 15311339

[2] Meuwly C Golanov E Chowdhury T Erne P Schaller B Trigeminal cardiac reflex: new thinking model about the definition based on a literature review Medicine 2015 94 5 e484 10.1097/MD.0000000000000484 25654391 PMC4602726

[3] Lübbers HT Zweifel D Grätz KW Kruse A Classification of potential risk factors for trigeminocardiac reflex in craniomaxillofacial surgery Journal of Oral and Maxillofacial Surgery 2010 68 6 1317 1321 10.1016/j.joms.2009.12.039 20347202

[4] Brunelli M Coppi E Tonlorenzi D Del Seppia C Lapi D Prolonged hypotensive and bradycardic effects of passive mandibular extension: evidence in normal volunteers Archives Italiennes de Biologie 2012 150 4 231 237 23479456 10.4449/aib.v150i4.1420

[5] Lapi D Colantuoni A Del Seppia C Ghione S Tonlorenzi D Persistent effects after trigeminal nerve proprioceptive stimulation by mandibular extension on rat blood pressure, heart rate and pial microcirculation Archives Italiennes de Biologie 2013 151 1 11 23 23807620 10.4449/aib.v151i1.1470

[6] Schaller B Probst R Strebel S Gratzl O Trigeminocardiac reflex during surgery in the cerebellopontine angle Journal of Neurosurgery 1999 90 2 215 220 10.3171/jns.1999.90.2.0215 9950491

[7] Lemaitre F Chowdhury T Schaller B The trigeminocardiac reflex -a comparison with the diving reflex in humans Archives of Medical Science 2015 11 2 419 426 10.5114/aoms.2015.50974 25995761 PMC4424259

[8] Lindholm P Sundblad P Linnarsson D Oxygen-conserving effects of apnea in exercising men Journal of Applied Physiology 1999 87 6 2122 2127 10.1152/jappl.1999.87.6.2122 10601158

[9] Del Seppia C Ghione S Foresi P Fommei E Lapi D Further evidence of a prolonged hypotensive and a bradycardic effect after mandibular extension in normal volunteers Archives Italiennes de Biologie 2016 154 4 143 150 10.12871/00039829201645 28306134

[10] Del Seppia C Ghione S Foresi P Lapi D Fommei Evidence in the human of a hypotensive and a bradycardic effect after mouth opening maintained for 10 min European Journal of Applied Physiology 2017 117 7 1485 1491 10.1007/s00421-017-3643-8 28509954

[11] Lapi D Federighi G Fantozzi MP Del Seppia C Ghione S Trigeminocardiac reflex by mandibular extension on rat pial microcirculation: role of nitric oxide PLoS One 2014 9 12 e115767 10.1371/journal.pone.0115767 25551566 PMC4281058

[12] Lapi D Varanini M Colantuoni A Del Seppia C Ghione S Repeated mandibular extension in rat: a procedure to modulate the cerebral arteriolar tone Frontiers in Physiology 2017 8 625 10.3389/fphys.2017.00625 28912722 PMC5583213

[13] Del Seppia C Lapi D Ghione S Federighi G Sabatino L Evidence in hypertensive rats of hypotensive effect after mandibular extension Physiological Reports 2018 6 23 e13911 10.14814/phy2.13911 30548831 PMC6291740

[14] Sabatino L Costagli C Lapi D Del Seppia C Federighi G Renin-angiotensin system responds to prolonged hypotensive effect induced by mandibular extension in spontaneously hypertensive rats Frontiers in Physiology 2018 9 1613 10.3389/fphys.2018.01613 30498455 PMC6249415

[15] Sabatino L Federighi G Del Seppia C Lapi D Costagli C Thyroid hormone deiodinases response in brain of spontaneausly hypertensive rats after hypotensive effects induced by mandibular extension Endocrine 2021 74 1 100 107 10.1007/s12020-021-02684-3 33761105

[16] Del Seppia C Federighi G Fommei E Ghione S Scuri R Hypotensive effect induced by mandibular extension in aged, hypertensive humans and rats Dental Oral Biology and Craniofacial Research 2021 1 5 10.31487/j.DOBCR.2021.01.06

[17] Arasho B Sandu N Spiriev T Prabhakar H Schaller B Management of the trigeminocardiac reflex: facts and own experience Neurology India 2009 57 4 375 10.4103/0028-3886.55577 19770535

[18] Lang S Lanigan DT van der Wal M Trigeminocardiac reflexes: maxillary and mandibular variants of the oculocardiac reflex Canadian Journal of Anaesthesia 1991 38 6 757 760 10.1007/BF03008454 1914059

[19] Gorini C Jameson HS Mendelowitz D Serotonergic modulation of the trigeminocardiac reflex neurotransmission to cardiac vagal neurons in the nucleus ambiguus Journal of Neurophysiology 2009 102 3 1443 1450 10.1152/jn.00287.2009 19553488 PMC2746775

[20] Gorini C Philbin K Bateman R Mendelowitz D Endogenous inhibition of the trigeminally evoked neurotransmission to cardiac vagal neurons by muscarinic acetylcholine receptors Journal of Neurophysiology 2010 104 4 1841 1848 10.1152/jn.00442.2010 20719927 PMC2957459

[21] Esser MJ Pronych SP Allen GV Trigeminal-reticular connections: Possible pathways for nociception-induced cardiovascular reflex responses in the rat The Journal of Comparative Neurology 1998 391 4 526 544 10.1002/(SICI)1096-9861(19980222)391:4<526::AID-CNE8>3.0.CO;2-2 9486829

[22] Rokx JT Jüch PJ van Willigen JD Arrangement and connections of mesencephalic trigeminal neurons in the rat Acta Anatomica 1986 127 1 7 15 10.1159/000146233 3024445

[23] Raappana P Arvidsson J Location, morphology, and central projections of mesencephalic trigeminal neurons innervating rat masticatory muscles studied by axonal transport of choleragenoid-horseradish peroxidase The Journal of Comparative Neurology 1993 328 1 103 114 10.1002/cne.903280108 8429123

[24] Kubota K Narita N Ohkubo K Hosaka K Nagae K Central projection of proprioceptive afferents arising from maxillo-facial regions in some animals studied by HRP-labeling technique Anatomischer Anzeiger 1988 165 2–3 229 251 3400886

[25] Lapi D Varanini M Galasso L Di Maro M Federighi G Effects of mandibular extension on pial arteriolar diameter changes in glucocorticoid-induced hypertensive rats Frontiers in Physiology 2019 10 3 10.3389/fphys.2019.00003 30792661 PMC6375092

[26] Goadsby PJ Duckworth JW Effect of stimulation of trigeminal ganglion on regional cerebral blood flow in cats The American Journal of Physiology 1987 253 2 Pt 2 R270 4 10.1152/ajpregu.1987.253.2.R270 3497587

[27] Drake CT Iadecola C The role of neuronal signaling in controlling cerebral blood flow Brain and Language 2007 102 2 141 152 10.1016/j.bandl.2006.08.002 17010421

[28] Cox SB Woolsey TA Rovainen CM Localized dynamic changes in cortical blood flow with whisker stimulation corresponds to matched vascular and neuronal architecture of rat barrels Journal of Cerebral Blood Flow and Metabolism 1993 13 6 899 913 10.1038/jcbfm.1993.113 8408316

[29] Goadsby PJ Knight YE Hoskin KL Butler P Stimulation of an intracranial trigeminally-innervated structure selectively increases cerebral blood flow Brain Research 1997 751 2 247 252 10.1016/s0006-8993(96)01344-3 9099811

[30] Ichinohe T Agata H Aida H Kaneko Y Cerebral cortex regional blood flow and tissue oxygen tension during the trigeminal depressor response in rabbits Journal of the Autonomic Nervous System 1997 66 1–2 111 118 10.1016/s0165-1838(97)00076-3 9335001

[31] White TG Powell K Shah KA Woo HH Narayan RK Li C Trigeminal Nerve Control of Cerebral Blood Flow: A Brief Review Frontiers in Neuroscience 2021 15 649910 10.3389/fnins.2021.649910 33927590 PMC8076561

[32] Sandu N Spiriev T Lemaitre F Filis A Schaller B New molecular knowledge towards the trigemino-cardiac reflex as a cerebral oxygen-conserving reflex The Scientific World Journal 2010 10 811 817 10.1100/tsw.2010.71 20454763 PMC5763844

[33] Golanov EV Reis DJ Neurons of nucleus of the solitary tract synchronize the EEG and elevate cerebral blood flow via a novel medullary area Brain Research 2001 892 1 1 12 10.1016/s0006-8993(00)02949-8 11172744

[34] Golanov EV Ruggiero DA Reis DJ A brainstem area mediating cerebrovascular and EEG responses to hypoxic excitation of rostral ventrolateral medulla in rat The Journal of Physiology 2000 529 Pt 2 (Pt 2) 413 429 10.1111/j.1469-7793.2000.00413.x 11101651 PMC2270200

[35] Moskowitz MA Wei EP Saito K Kontos HA Trigeminalectomy modifies pial arteriolar responses to hypertension or norepinephrine The American Journal of Physiology 1988 255 1 Pt 2 H1 6 10.1152/ajpheart.1988.255.1.H1 3394814

[36] Chiluwal A Narayan RK Chaung W Mehan N Wang P Neuroprotective effects of trigeminal nerve stimulation in severe traumatic brain injury Scientific Reports 2017 7 1 6792 10.1038/s41598-017-07219-3 28754973 PMC5533766

[37] Li C Chiluwal A Afridi A Chaung W Powell K Trigeminal nerve stimulation: a novel method of resuscitation for hemorrhagic shock Critical Care Medicine 2019 47 6 e478 e484 10.1097/CCM.0000000000003735 30889027

[38] Leeuwis AE Smith LA Melbourne A Hughes AD Richards M Cerebral Blood Flow and Cognitive Functioning in a Community-Based, Multi-Ethnic Cohort: The SABRE Study Frontiers in Aging Neuroscience 2018 10 279 10.3389/fnagi.2018.00279 30279656 PMC6154257

